# Is there a correlation between male partner’s previous tuberculosis and recurrent pregnancy loss (RPL)? – a single-center retrospective analysis

**DOI:** 10.1515/med-2025-1370

**Published:** 2026-02-12

**Authors:** Xin-Zhuan Jia, Lan Wei, Bo-lin Zheng, Er-huan Liu, Li-na Guo, Na Zhang

**Affiliations:** Department of Reproductive Medicine, The Fourth Hospital of Hebei Medical University, Shijiazhuang, Hebei Province, China; Chest Surgery, Hebei Chest Hospital, Shijiazhuang, Hebei Province, China; Women’s Health Guidance Department, Tianjin Hedong District Maternal and Child Health and Family Planning Service Center, Tianjin, China; Operating Room, Shenzhou People’s Hospital, Shenzhou, Hebei Province, China

**Keywords:** tuberculosis, recurrent pregnancy loss, sperm DNA fragmentation index, testosterone

## Abstract

**Objectives:**

The research on idiopathic recurrent pregnancy loss (RPL) mainly focuses on women, but recently researchers began to explore the potential contribution of male partners. Growing evidence suggests that male tuberculosis may contribute to adverse pregnancy outcomes. We studied whether men’s previous tuberculosis may contribute to idiopathic RPL in early pregnancy.

**Methods:**

A retrospective study on 182 couples with idiopathic RPL in early pregnancy (study group) and 260 couples with fertility (control group) who visited the Fourth Hospital of Hebei Medical University from January 1, 2021 to December 31, 2022 was conducted. Logistic regression analysis was performed to nvestigate the correlation between male partner’s previous pulmonary tuberculosis and idiopathic RPL.

**Results:**

Male partners accounted for 37.91 % of previous pulmonary tuberculosis in the study group, and the sperm DNA fragmentation index (DFI) in the study group was higher than that in the control group (17.52 ± 7.87 vs. 7.79 ± 4.49, p=0.000). After adjusting for factors, Logistic regression analysis showed that male partners with previous tuberculosis history were prone to RPL (p=0.000), and male partners with untreated tuberculosis history were more prone to idiopathic PRL [treated tuberculosis history vs. untreated tuberculosis (OR 29.557, 95 % CI 6.437–135.708, p=0.000), no tuberculosis history vs. untreated tuberculosis history (OR 73.856, 95 % CI 13.139–415.150, p=0.000)]. Spearman correlation showed that the prevalence of tuberculosis in male partners was positively correlated with DFI (R=0.492, p=0.000) and negatively correlated with testosterone (R=−0.120, p=0.012).

**Conclusions:**

Among the couples with idiopathic PRL, male partners with a history of pulmonary tuberculosis (especially untreated pulmonary tuberculosis) are more prone to PRL, which maybe caused by high DFI and low testosterone.

## Introduction

Recurrent pregnancy loss (RPL) is defined as two or more consecutive clinical pregnancy failures before 20 weeks. The etiology of RPL is complicated and multifactorial, and the root cause of RPL in about 40–50 % cases has not been determined [1], 2]. Recent studies have shown that sperm abnormalities may play a role in the pathophysiology of RPL [3]. The basis of male fertility survey is semen analysis. However, conventional semen analysis only provides information about sperm concentration, motility and morphology, but not functional information [4]. Compared with fertile couples, couples who have experienced RPL have higher sperm DNA fragmentation (SDF) [3], [5], [6], [7], [8]. Abnormal DFI can be considered as one of the potential factors leading to RPL. For cases with a history of RPL, sperm DNA fragmentation should be studied as part of routine semen analysis [9].

At present, most studies pay attention to the influence of female tuberculosis on adverse pregnancy outcomes, and few studies pay attention to the role of male partner tuberculosis in on adverse pregnancy outcomes. Studies have shown that the possibility of stillbirth of women with tuberculosis is 2.13 times that of women without tuberculosis [10]. Other animal experiments have confirmed that non-disseminated pulmonary tuberculosis can affect the reproductive organs and sperm production of male mice [11]. In our study, we investigated whether the male partner’s previous tuberculosis may contribute to the primary idiopathic RPL.

## Materials and methods

### Research design

This is a retrospective cohort study. The study population is the couple with idiopathic RPL who visited the Fourth Hospital of Hebei Medical University in China from January 1, 2021 to December 31, 2022. Primary idiopathic RPL should meet the following conditions [3], 5], 12]: (1) There were no abnormality in the genetic analysis of pregnancy tissue and the karyotype analysis of parents; (2) Antiphospholipid antibodies [lupus anticoagulant (LA) and anticardiolipin antibodies (ACA IgG and IgM)], β2 glycoprotein I antibody (aβ2g p1) and antinuclear antibody (ANA) were all negative; (3) TSH and TPO-antibody were negative; (4) No abnormality was found in gynecological B-ultrasound examination; (5) Cervical-vaginal infection screening was negative; (6) Glucose metabolism was normal; (6) No live births. Inclusion criteria: (1) The male was under 45 years old and his spouse is between 25 and 35 years old; (2) The BMI of both husband and wife was between 18.5 and 23.9; (3) Both spouses had no malignant tumor, AIDS, cardiovascular diseases, metabolic diseases, autoimmune diseases and sexually transmitted diseases; (4) The cases of primary idiopathic RPL were considered as the study group; The couples with proven fertility (that is, who had a full-term pregnancy and live birth within 1 year before the study registration) were considered as the control group; (5) The case data was complete. Exclusion criteria: (1) Male and/or female had active tuberculosis; (2) Chromosome abnormality; (3) RPL after IVF-ET; (4) The woman with previous history of tuberculosis; (5) Sarcoidosis, other granulomatous diseases except tuberculosis, infection, tumor or occupation-related lung diseases were suggested by chest CT. The data needed for this study came from the case registration research database of the Fourth Hospital of Hebei Medical University.

**Figure 1:**
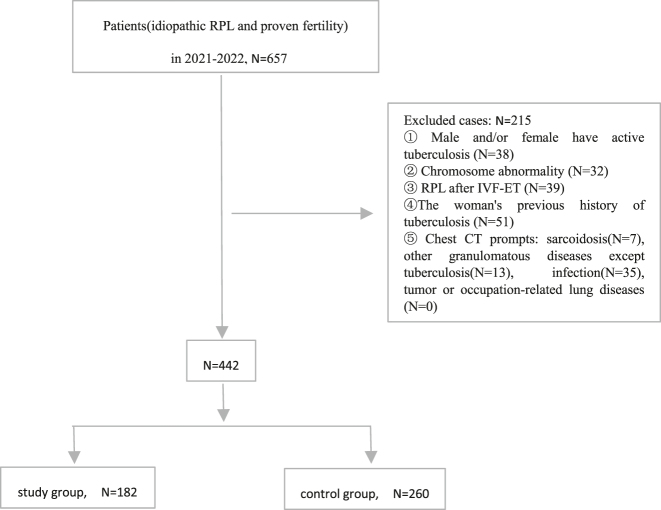
Study flow chart.

According to the inclusion criteria, 657 cases were collected According to the exclusion criteria, 215 cases were excluded. The study group collected 182 couples with idiopathic RPL. The control group collected 260 couples with fertility. Previous pulmonary tuberculosis (PTB) was defined as the radiological signs of old or inactive PTB [13], [14], [15], including discrete linear or reticular fibrosis scars of upper lung, sclerotic or calcified lesions and pleural thickening with/without calcification of hilar or mediastinal lymph nodes. This definition did not require a clear history of tuberculosis. Figure 1.

This retrospective analysis was conducted in accordance with the ethical norms of the World Medical Association (Helsinki Declaration) and was approved by the Ethics Committee of the Fourth Hospital of Hebei Medical University (approval number: 2021KS010). Informed consent was not required.

### Semen analysis

Collect and process semen specimens in accordance with the standards of the 5th edition of the World Health Organization Manual for Human Semen Testing and Processing Laboratories [16]. After abstinence for 2–5 days, semen samples were obtained by masturbation and stored in sterile containers. After semen liquefaction, routine parameters of sperm (including total sperm count, sperm concentration, and progressive motility) were analyzed using a computer-aided semen analysis operating system (CASA system, Micropic, Spain). Sperm were stained with Diff-Quik staining solution (Shenzhen Kanghua Biology, China), and the morphological characteristics of sperm were manually analyzed under an optical microscope. DFI and high-dye sperm index (HDS) were determined by the Sperm Chromatin Structure Analysis (SCSA). After staining the sperm with the sperm nuclear integrity staining kit (Anhui Anke Biology, China), the samples were analyzed using flow cytometry (Shenzhen Mindray, China). Subsequently, the DFI and HDS were evaluated using the FCSAS software.

### Statistical analysis

SPSS.20 was used for statistical analysis. Continuous variables were expressed as mean ± standard deviation (SD) and analyzed by independent-sample *t*-tests. The classified variables were expressed as n (%), and were analyzed by chi-square test. Logistic regression was used to evaluate the influence of exposure factors on RPL. Spearman rank correlation analysis was applied for bivariate linear correlation analysis. A two-tailed p-value of <0.05 was considered statistically significant.

## Results

### Characteristics of two groups

The age of couples and the prevalence of tuberculosis in males were higher in the study group than those in the control group (p<0.05). The serum testosterone levels in males in the study group were lower than those in the control group (p<0.05). Table 1.

**Table 1:** Characteristics of two groups.

Characteristics	Study group (n=182)	Control group (n=260)	χ^2^/*t*	p-Value
Male				

Age, years (mean ± SD)	34.52 ± 4.90	32.86 ± 5.31	3.34	0.001
BMI, kg/m^2^ (mean ± SD)	22.03 ± 2.07	22.32 ± 2.46	1.32	0.189
Smoke, n (%)			1.83	0.176
No	52.75 % (96/182)	59.23 % (154/260)		
Yes	47.25 % (86/182)	40.77 % (106/260)		
Drink wine/alcohol, n (%)			0.39	0.535
No	55.49 % (101/182)	58.46 % (152/260)		
Yes	44.51 % (81/182)	41.54 % (108/260)		
Previous history of tuberculosis, n (%)			21.76	0.000
No	62.09 % (113/182)	81.92 % (213/260)		
Yes	37.91 % (69/182)	18.08 % (47/260)	6.307	0.012
Untreated	33.33 % (23/69)	12.77 % (6/47)		
Treatment	66.67 % (46/69)	87.23 % (41/47)		
Hormone level, (mean ± SD)				
FSH, mIU/mL	6.35 ± 1.59	6.49 ± 1.50	0.997	0.319
E_2_, pg/mL	40.53 ± 9.82	39.29 ± 8.02	1.45	0.147
T, ng/mL	3.59 ± 0.71	3.80 ± 0.94	2.62	0.009

Female				

Age, years (mean ± SD)	30.12 ± 5.07	31.60 ± 4.13	3.37	0.001
BMI, kg/m^2^ (mean ± SD)	22.17 ± 2.04	22.29 ± 2.06	0.59	0.559
Smoke, n (%)			0.02	0.903
No	91.21 % (166/182)	91.54 % (238/260)		
Yes	8.79 % (16/182)	8.46 % (22/260)		
Drink wine/alcohol, n (%)			0.13	0.742
No	73.08 % (133/182)	74.62 % (194/260)		
Yes	26.92 % (49/182)	25.38 % (66/260)		

BMI, body mass index; FSH, follicle-stimulating hormone; E_2_, estradiol; T, testosterone.

### Comparison of semen quality, DFI and HDS between the two groups

The DFI in the study group was significantly higher than that in the control group (p=0.001), and there was no difference in HDS and sperm quality between the two groups (p>0.05). Table 2.

**Table 2:** Male partners in semen quality, DFI and HDS of 2 groups.

Baseline level	Research group	Control group	*t*	p-Value
DFI, % (mean ± SD)	17.52 ± 7.87	7.79 ± 4.49	16.47	0.000
HDS, % (mean ± SD)	7.53 ± 3.81	7.76 ± 3.80	0.64	0.525
Sperm quality (mean ± SD)				
Sperm concentration, ×10^6^/mL	64.22 ± 24.93	67.68 ± 27.49	1.35	0.177
Progressive motility, %	63.66 ± 17.37	62.22 ± 17.05	0.87	0.385
Total sperm count, ×10^6^	139.31 ± 55.91	147.26 ± 56.09	1.47	0.143
Normal sperm morphology rate, %	5.82 ± 1.18	6.04 ± 1.22	1.89	0.060

DFI, sperm DNA, fragmentation index; HDS, high-dye sperm index.

### Influence of exposure factors on RPL

Logistic regression showed that male age, T, DFI and previous tuberculosis history had significant effects on RPL (p<0.05). Table 3. After adjusting the above factors, men with a history of previous tuberculosis were prone to RPL (p=0.000), and male partners with untreated tuberculosis history were more likely to have idiopathic PRL [treated tuberculosis history vs. untreated tuberculosis (OR 29.557, 95 % CI 6.437–135.708, p=0.000), no tuberculosis history vs. untreated tuberculosis history (OR 73.856, 95 % CI 13.139–415.150, p=0.000)].

**Table 3:** Influence of exposure factors on recurrent prenancy loss.

Covariates	Wald test	p-Value	OR (95 % CI)
Male partner			

Age	4.194	0.041	1.058 (1.002–1.116)
BMI	3.579	0.059	1.051 (0.913–1.110)
FSH	1.043	0.307	0.908 (0.755–0.967)
E_2_	1.836	0.175	0.977 (0.945–1.010)
T	6.142	0.013	0.629 (0.436–0.908)
DFI	95.952	0.000	1.487 (1.374–1.610)
HDS	1.540	0.215	0.953 (0.883–1.028)
Sperm concentration	1.641	0.200	0.993 (0.982–1.004)
Forward motion	0.295	0.587	0.995 (0.979–1.012)
Total sperm count	0.510	0.475	0.998 (0.993–1.003)
Normal sperm morphology rate	0.977	0.323	0.889 (0.704–1.122)
Smoking history	0.976	0.322	0.734 (0.397–1.356)
Drinking history	1.415	0.234	0.683 (0.364–1.280)
History of tuberculosis	34.257	0.000	
Non-TB and treated TB	32.257	0.000	17.639 (9.948–21.589)
Non-TB and untreated TB	17.447	0.000	25.210 (5.545–65.614)

Female partner			

Age	1.740	0.187	0.960 (0.904–1.020)
BMI	0.001	0.972	0.998 (0.872–1.141)
Smoking history	2.436	0.119	2.280 (0.810–6.419)
Drinking history	0.024	0.878	0.952 (0.507–1.788)

BMI, body mass index; FSH, follicle-stimulating hormone; E_2_, estradiol; T, testosterone; DFI, sperm DNA, fragmentation index; HDS, high-dye sperm index.

### Correlation analysis between the prevalence rate of previous tuberculosis in men, and the DFI, testosterone

The prevalence rate of previous tuberculosis in men was positively correlated with DFI (R=0.492, p=0.000) and negatively correlated with testosterone (R=−0.120, p=0.012).

## Discuss

For a long time, it has been thought that the factors affecting the development of embryos and fetuses are entirely from the maternal line; Therefore, if there are problems related to fertility and embryo development, traditionally, it can only be blamed on the mother. However, studies have begun to prove that this is not the case. Sperm not only carries the paternal haploid genome to the oocyte, but also continues to play various roles in the embryonic development [17]. In RPL, pregnancy is not a problem, but the pregnancy of live births is a problem, and the role of male factors is highly ignored after fertilization. In our retrospective analysis, the male partners in primary idiopathic PRL couples were taken as the research object, and it was found that the prevalence of tuberculosis in male partners was higher than that in the control group, and the serum testosterone levels were lower than those in the control group. The prevalence of tuberculosis in the past was positively correlated with DFI and negatively correlated with testosterone levels. The decrease of serum testosterone levels was found in male tuberculosis patients with different degrees, which may play a role in the course of tuberculosis [18]. It is reported that the serum testosterone concentration of male patients suffering from tuberculosis [18], leprosy [19] and neuropool disease decreased significantly [20]. Non-disseminated pulmonary tuberculosis affects male reproductive organs and sperm production, which is due to hormonal changes, imbalance of pro-inflammatory cytokine spectrum and disease wasting syndrome [11].

The preliminary investigation of RPL male partners mainly focuses on routine semen analysis, including the evaluation of sperm concentration, motility, vitality and morphology. Abnormal sperm motility or other semen parameters are related to changes in sperm function, which can be used as a reference factor or risk factor in RPL. However, only having normal semen parameters can not ensure normal sperm function, so it becomes an inaccurate method to predict pregnancy outcome [21]. The sperm of RPL male partners showed a high proportion of sperm apoptosis, sperm deficiency and DNA breakage [22], 23]. Abnormal DFI can be considered as one of the potential factors leading to RPL. For cases with a history of recurrent abortion, sperm DNA fragmentation should be studied as part of routine semen analysis [9]. A systematic review in 2019 included 15 studies on sperm DNA damage and RPL, and found that the rate of sperm DNA damage of RPL male partners was significantly higher than that of male partners in fertile couples [6]. It is consistent with the results of this study.

In our research, it is confirmed that male partners with a previous history of pulmonary tuberculosis are prone to PRL. Sperm containing SDF successfully fertilized the oocyte, but if the oocyte failed to repair all the damage, the residual SDF might be passed on to the fertilized egg. High SDF is related to low embryo quality [24], 25] and abortion [26]. In animal experiments [11], compared with healthy mice, the serum testosterone of mice infected with *Mycobacterium tuberculosis* only showed a downward trend, and the weight of their testes did not change, and no histopathological changes were found. However, a significant imbalance of cytokine spectrum was observed in the testes (the levels of IL-6 increased, IL-10 and TGF-b decreased), which triggered changes in testicular function. The change of cytokine expression found in the testis of animals infected with *M. tuberculosis* can be explained by the influence of systemic inflammation on testicular macrophages [27]. Upon infection with *M. tuberculosis*, host macrophages undergo potent activation, which results in excessive production of reactive oxygen species (ROS) and several proinflammatory mediators. At physiological concentrations, ROS act as molecular mediators of signal transduction pathways involved in the regulation of the hypothalamic-pituitary-gonadal axis, spermatogenesis and steroidogenesis. ROS are potent signalling intermediates produced in response to GnRH stimulation and influence the gonadotrope response by targeting the MAPK cascade [28], excess ROS exert cytotoxic effects and can disrupt signalling pathways. Oxidative stress can have deleterious effects on DNA integrity and an increased rate of sperm DNA fragmentation has been reported in infertile patients having high levels of ROS [29].

Our study found that the proportion of male partners in the study group who had not been treated with tuberculosis was higher than that in the control group. Male partners with untreated tuberculosis history were more likely to have primary idiopathic PRL. This can be explained by the fact that TB-specific cellular immunity may persist after untreated TB is spontaneously cured [30], 31].

However, there are still some limitations in this study. First of all, this study only conducted this logistic regression analysis without combining other statistical methods to verify the robustness of the results, which may affect the reliability of the conclusions. In addition, The sample too small and limited to untreated tuberculosis cases, which limits the universality of the results. Secondly, other demographic or sociological factors, such as education, economic situation and working environment, may also affect sperm DFI, but we don’t have these available data. In addition, multivariate logic analysis is used in our research, and the results are still indicative and need to be verified in larger queues and prospective studies.

## Conclusions

In a word, our study have found that male partners with a history of tuberculosis (especially untreated tuberculosis) are more prone to PRL, which may be caused by high DFI and low testosterone.
